# Obsessionality & compulsivity: a phenomenology of obsessive-compulsive disorder

**DOI:** 10.1186/1747-5341-6-3

**Published:** 2011-02-01

**Authors:** Damiaan Denys

**Affiliations:** 1 Department of Psychiatry, Academic Medical Center (AMC), University of Amsterdam, Amsterdam, the Netherlands; 2 Netherlands Institute for Neuroscience, an institute of the Royal Netherlands Academy of Arts and Sciences, Amsterdam, the Netherlands

## Abstract

Progress in psychiatry depends on accurate definitions of disorders. As long as there are no known biologic markers available that are highly specific for a particular psychiatric disorder, clinical practice as well as scientific research is forced to appeal to clinical symptoms. Currently, the nosology of obsessive-compulsive disorder is being reconsidered in view of the publication of DSM-V. Since our diagnostic entities are often simplifications of the complicated clinical profile of patients, definitions of psychiatric disorders are imprecise and always indeterminate. This urges researchers and clinicians to constantly think and rethink well-established definitions that in psychiatry are at risk of being fossilised. In this paper, we offer an alternative view to the current definition of obsessive-compulsive disorder from a phenomenological perspective.

**Translation:**

This article is translated from Dutch, originally published in [Handbook Obsessive-compulsive disorders, Damiaan Denys, Femke de Geus (Eds.), (2007). De Tijdstroom uitgeverij BV, Utrecht. ISBN13: 9789058980878.]

## A case of obsessive-compulsive disorder

At consultation a young mother explains that she constantly thinks about strangling her daughter. The thought occurred without any apparent cause shortly after the birth of her daughter and is always present. *"When I'm alone at home and I see my daughter sleeping in her crib then I can see myself strangling her. I'm terribly shocked by the thought and I am very frightened by it. If nobody holds me back, I could murder my daughter. I don't want to harm her, but there is no guarantee that I never will. I can't control myself any longer. I thought I was a good mother, but the fact that I think about it says something about who I really am. It shows that perhaps I don't love my daughter enough. I don't want to think about it but I'm not able to keep the thought out of my mind. The harder I resist, the stronger the thought is. In the beginning I occasionally thought about it, but now I think about it all the time. Though I realize that the thought is absurd, I can't stop it" *The patient is deeply embarrassed and feels very guilty. Nobody knows that she has these thoughts, not even her husband. Given that she does not trust herself she does not want to be alone with her daughter. *"Whenever the thought occurs, I wash my hands. It seems that I can rinse this terrible thought away causing the anxiety to decrease. I have to do it exactly 8 times and am not allowed to touch anything else while washing or to think about my daughter. If I don't do it properly, I have to start all over again. It's becoming increasingly worse. Now I wash my hands twelve times a day for about twenty-five minutes. At the moment I find washing my hands more annoying than the thought itself. It takes a long time and I become very anxious when I don't have the facilities to wash my hands."*

## Deconstructing obsessions and compulsions

In this prototypical case, the symptoms that are described in classic textbooks as obsessions and compulsions are easy to recognise. The intrusive thought or the obsession of the young mother that she could strangle her daughter is a recurring and persistent idea that causes anxiety or unease, and hand-washing or the compulsion is a ritualistic act that is stereotypically performed to reduce the anxiety caused by the obsession. Patients with obsessive-compulsive disorder (OCD) recognise the unreasonable and excessive nature of their obsessions and compulsions. Even though this classic definition appears to give a clear description of OCD, it is problematic in several aspects.

## Phenomenology

In order to offer an alternative perspective to the current definition, this paper will inquire into OCD from a phenomenological perspective. Although phenomenology in psychiatry is synonymous with the pure clinical description of symptoms, phenomenology aims to stay ahead of the symptoms and, as open minded as possible, freed from theoretical assumptions and premises, to sketch a picture of the patients' immediate and original mental experience. "*Phenomenology is the study of essences; and according to it, all problems amount to finding definitions of essences: the essence of perception, or the essence of consciousness, for example. Phenomenology tries to give a direct description of our experience as it is, without any reference to its psychological origins and to the causal statements made by the scientist, the historian or the sociologist. Phenomenology means a return to the things themselves: a return to a world prior of knowledge in which knowledge always speaks and from which any scientific determination is abstract, indicative and dependent, such as geography in relation to landscape" *[1]. The term "essence" derives from the Greek *ousia*, which means the inner essential nature of a thing, the true being of a thing. The Latin *essentia*, from *esse *means "to be." Essence is that what makes a thing what it is (and without which it would not be what it is); that what makes a thing what it is rather than its being or becoming something else. In phenomenology, Essence is a complex notion that alludes to the ways that a phenomenon reveals itself in thinking, to the ways that we encounter something, and to the ways that we ourselves are constantly put into question by the being of the things of our world. The term essence does not describe the whatness of a phenomenon but it describes the meaning relations that we maintain with the world. Essence is a relational term that refers to the intentionalities of our world, to possible ways of encountering and relating to the things of our world before and while we understand or think them in language and poetic and conceptual thought [2]. For Husserl phenomenology is a discipline that endeavors to describe how the world is constituted and experienced through conscious acts. His phrase *Zu den Sachen *means both "to the things themselves" and "let's get down to what matters!" Phenomenology must describe what is given to us in immediate experience without being obstructed (mediated) by pre-conceptions and theoretical notions [2]. This paper aims to describe the essence of OCD, in an anecdotal manner without reference to theoretical models but with regular reference to the clinical stories of patients themselves. The importance of phenomenology is not to formulate a conclusive answer but to produce new questions to highlight different perspectives of the disorder. Because phenomena are transient, a phenomenological approach always will be provisional and therefore ongoing. Its value is in sketching new angles.

## Obsessionality

The first element that strikes the reader/observer in the above-mentioned case is the obsessionality of the patient. Obsessionality shall be used here in its original meaning, derived from the Latin word "obsidere": being taken into possession, being occupied or preoccupied. The patient is obsessed with the idea that she could harm her daughter but also with the ritual act of washing her hands. She is focused on a single theme and is completely seized by it. According to phenomenology, every mental act has a particular intentionality, a certain orientation and direction towards content. Observing, thinking, desiring are focused on something that is observed, thought or desired. The term intentionality expresses the relationship between mental acts and inner or outer contents. The way the patient in this vignette relates to themes in her thought and act is clearly different from her orientation to everyday things. The relationship to the theme is more focused, more powerful, longer and more specific. Her attention is focused on a single thought or action and is held on to longer. In obsessionality the "normal" intentionality of mental acts is disturbed.

Contrary to what the term suggests, obsessionality does not specifically relate to obsessions. Obsessionality is recognisable in thoughts and acts, in obsessions and compulsions. The patient is obsessed with the idea that she could strangle her daughter but also with the act of washing hands. In both cases there is a specific and direct aim that totally occupies her. However, obsessionality differs from the thought and from the act. The intrusive thought (obsession) causes the patient to feel that she is passively subjected to the predominant overruling nature of the thought. In the formal act (compulsion), the patient actively seeks focused attention to perform the ritual act as precisely as possible. The patient in the above-mentioned case *is being obsessed *by the thought that she could strangle her daughter, but *is obsessed *with the act of washing hands. In the intrusive thought, the patient's attention to the world is deprived and is selectively focused on a topic; and in the compulsive act, the patient diverts her attention deliberately from the world to selectively focus on a topic. During a compulsive act, the patient intentionally wants to be fully aware of every detail of its execution because a ritual only works if it is performed perfectly, if one is fully focussed, when one is fully seized by it. During the obsession the patient's attention for the world is shut off and is focused selectively on one subject, during the compulsion it is the patient who shuts its attention off from the world to focus selectively on one subject. One may speak of *"passive obsessionality" *during obsessions because the patient is submissively being obsessed and of *"active obsessionality" *during compulsions because the patient actively takes something into obsession. This difference is easy recognisable in the pattern of complaints of our case. The patient perceives being possessed by the thought that she could strangle her daughter as very disturbing, but when she is washing her hands she perceives the world and the others who distract her from her ritual act as disturbing. To turn away from the world to have to think about the intrusive thought is a source of annoyance, but so is to have to turning to the world when a compulsive ritual act is interrupted.

Obsessionality or obsessive intentionality goes hand in hand with a number of properties that are very recognisable in OCD. The world is unable to reveal itself because one is unable to take enough distance from the intentional object. There is only that one thought or one act. In obsessionality, the obsessed subject coincides with the object of obsession, nearly melts together with the object so that the rest of the world is meaningless and even threatens to vanish. This puts the patient with OCD inevitably into an affective and cognitive isolation. A real relation with the world is no longer possible. Apart from the instrumental role in which the world and the others are placed, serving the purpose of the ritual, the patient suffering OCD lives a solitary existence. Since the overall perspective has been lost, the reference frame of "normality" also vanishes. The patient is unable to judge within obssesionality the exaggerated and unreasonable nature of his symptoms adequately. Obsessionality is by definition irrational because in dealing with the details the notion of the whole is missed. One can check the lock of the door for hours out of fear for burglary and at the same time fail to notice the attic window that is ajar, or endlessly clean the bathroom sink because of contamination fear while ignoring the lavatory. The impossibility to put the importance of obsessions and compulsions into perspective is central and inherent to obsessionality.

In obsessionality the patient not only runs the risk of losing the world and the other, but also himself. Many patients feel they only exist by the grace of their attention to the obsessional theme. Patients suffering OCD want to be fully aware of every mental act in order not to lose themselves, not to submerge. The Cartesian "Cogito, ergo sum", I think, therefore I am, or rather the negation of it, when I do not think, therefore I am not, is literally expressed in obsessionality. The patient with OCD is afraid to disappear as a subject, to submerge in the void when the world is not consciously handled. The self-conscious and explicit way of handling reality to acquire continuous proof of its existence creates a dependence on the conscious act, making giving in to reality an even bigger challenge for patients suffering from OCD. In OCD, self-consciousness paradoxically leads to the inability to merely accept reality, to cope with the world, since it increasingly separates the patient suffering from OCD further from the world, causes even more anxiety and forces the patient even deeper into self-consciousness. By consciously managing the world, the OCD patient determines what will be seen and not what will be shown. OCD leaves the world and the other without a chance. It is quite understandable that in the self-conscious relationship with the world the OCD patient lives under the impression that responsibility only rests on his shoulders while it otherwise is borne by the world and other people. Typically, in obsessive compulsive disorder, patients are convinced that they solely are responsible for dangers, car accidents, contamination, etc. One patient, for example, saw a big boulder hanging above a small river near to a camping site where children used to play. He was obsessed with the idea that the boulder might fall, and that he would be responsible because he knew of the danger and no one else. Seeing things make patients with OCD immediately responsible for those things.

A corollary of obsessionality, and of particular interest for its neurobiological underpinning, is the process of reinforcement and association. The more we focus on a subject, the faster it occurs to us and the easier it is associated with another subject. We know that our brain ultimately takes the shape of the activity we exercise, as expressed in the saying "Practice makes perfect", "Our nervous system grows to the modes in which it has been exercised", "What wires together fires together" or "La fonction fait l'organe". Obsessive-compulsive symptoms can only increase, despite resistance of the patient, because resistance to an obsessional theme still is part of obsessionality of the theme. It is likely that the process of reinforcement and association that parallels obsessionality is based on normal neurobiological mechanisms such as the principle of neuronal plasticity, which is indispensable for any learning process. Given that reinforcement and association are inherent to focused attention, it is perhaps surprising that more people are not obsessional. The question is not why OCD exists, but why it does not exist more?

Obsessionality is indispensable for defining OCD but not specific to OCD. Obsessionality is also present in other psychiatric disorders such as delusions, anorexia and body dysmorphic disorder. In normality, obsessionality also occurs. Elite athletes, musicians, chess-players, writers, artists, politicians, scientists, many of them are preoccupied with one specific theme or goal. Here we find the same characteristics of being possessed and the same mechanism of exclusive attention with reinforcement and association. None of these characteristics are thus pathological by themselves. It is not clear whether obsessionality in OCD differs in other aspects from obsessionality in psychiatric disorders and from obsessionality in normality. Is it based on other underlying neurocognitive principles? Is there another neurobiological mechanism involved or is it the same mechanism that is dysfunctional in OCD? Is the distinction based on the purpose of obsessionality? Can political, scientific or artistic obsessionality be enduring and normal for it is founded on a personal conviction, because it has a social value, or because it is not seen as threatening? Obsessionality is an essential feature of OCD and necessary to describe the disorder, but insufficient to define it from other disorders or normality.

## Compulsivity

Another significant element in the story of the patient is compulsivity. Compulsivity is also used here in its original meaning, derived from Latin "compellere": feeling forced to or being cornered. The patient in the above-mentioned case is not only obsessed by a theme, she also feels forced to be occupied with it despite fruitless efforts not to be. She does not feel free in what she thinks and what she does. She feels that something in her determines what to think and how to act. She has lost control of herself.

Compulsivity exists in an objective and a subjective variety. Objective compulsivity is the mechanism through which one mental event necessarily leads to another mental event. It is the principle of determination: a certain mental act leads inevitably to another mental act. A depressed mood forces to gloomy thoughts; a joyous mood forces cheerful behaviour. The concept of compulsivity serves well here, because it is assumed that under the given circumstances it is impossible for a person to react differently than is prescribed. The sequence of certain mental events may well be determined, yet, one does not necessarily have to realize or notice this. Subjective compulsivity indicates the *feeling *of being compelled. Thinking of the obsession and exercising the compulsion gives the patient with OCD the impression of being forced exactly to think this and do this. Objective compulsivity is seen in various disorders and compulsivity but subjective compulsivity is typical of OCD.

Compulsivity, like obsessionality, is recognisable in both obsessions and compulsions. The patient in the abovementioned case feels compelled to think of strangling her daughter, and she also feels compelled to wash her hands. Yet, here there is also a shade of difference between obsessions and compulsions in experiencing compulsivity. Compulsivity is immediately implied in obsessions but gradually materializes in compulsions. The patient experiences the thought of strangling her baby immediately as intrusive and compulsive. However, the washing of her hands, or at least so it seems at the beginning of the process of the illness, is a deliberate and free act, meant to regulate the fear originated from the obsessions, and only becomes compulsive after some time. In the beginning, washing her hands helps her to calm down and the act is relatively limited, but later on the washing increases to such an extent that the impossibility of washing hands causes fear. The forced nature of the compulsive act adheres to the pattern of addiction. Originally, the act is functional and effective (cleaning helps to reduce anxiety). Later on, the compulsion increasingly expands because of the tolerance to the effect (one needs to clean at least twenty times to reduce anxiety feelings instead of a few times). Gradually, the compulsion loses its effectiveness (cleaning does not help anymore to reduce anxiety). Ultimately, the compulsion may not be omitted anymore to avoid intense distress and its manifest consequences in many- if not all- dimensions of the patient's life (cleaning is needed just to live). The process of compulsivity that develops in a compulsion resembles the process of compulsivity in addiction disorders. In an obsession, compulsivity is *direct*, because it is experienced from the beginning and it coincides with obsessionality, in a compulsion, compulsivity is *indirect *because it occurs only after a lap of time and is the result of a typical process. Apart from that, it is not clear whether the patient with OCD has the freedom to choose a compulsion. Could the patient in the abovementioned case have exercised another ritual or was she forced to washing her hands? What determines the choice of a compulsion?

Compulsivity raises the problem of free will. A patient with OCD literally feels forced to think a thought or to perform an act. Since there is no arbitrary possible choice that can be made, there is no sense any longer of free will. The absence of free will goes side by side with the sense of lack of control and the loss of control gives reason to fear, because one has got the feeling of having lost the mastery over one's own thinking and behaving. Of importance is, that all the time it is about feeling or experience, not about the actual reality. An exceptional example from clinical practice illustrates how meaningful the distinction between a compulsion and free will is to patients suffering from OCD. A middle-aged man comes to our ward and complains about serious obsessions with a violent and sexual content. Images are forced upon him where his wife is murdered, and where explicit sexual scenes between men occur. The obsession frightens him a lot. Questioning him further, the man admits having a certain interest in violence and sexuality. He admits to have been occasionally actively looking on the Internet for sexually provocative and violent images. He also states that he suffers from intrusive thoughts in the morning and in the afternoon looks for similar images on the Internet. According to the man, there is no substantive difference with respect to the content of his obsessions and the images he is looking for on the Internet. Yet the obsession frightens him a lot because they are out of his control, whereas he feels safe with the images on the Internet because he can manipulate them. This exceptional case strikingly illustrates that almost simultaneously the same thematic content once experienced as aggravating because of its compulsive nature, will be enjoyed next time because of its voluntary nature.

Experiencing compulsivity on a daily base, OCD patients are equipped with a fine psychological sense for the subtle distinction between a free and a forced mental act. They experience an obsession as compulsive and therefore as involuntary and the resistance against the same obsession as an act of free will. As a matter of fact, the feeling of compulsivity can only be experienced in the context of free will, just like a lie is only possible in the context of the truth. Subjective compulsivity is only possible when the patient is capable of experiencing free will and that can be relevant for the differential diagnosis. In principle, the diagnosis of OCD cannot be made in patients with schizophrenia, a severe addiction, Parkinson's disease or Down syndrome. Patients suffering from these disorders are not free in their thinking or acting and thus the compulsivity of their thinking and acting cannot be experienced, as a result of which OCD cannot be diagnosed. This is a radical view, but cuts-off the compulsions in OCD from the meaningless stereotype, repetitive, ritualistic behaviours that often are observed in schizophrenia, addiction, mental retardation, or dementia. The strict premise of subjective compulsivity in OCD not only helps to distinguish compulsive from instinctive behaviour, but also compulsivity from impulsivity. In both compulsivity and impulsivity there is loss of control. In the case of compulsivity, the subjective compulsivity is foregrounded: there is a feeling of absolute loss of control while in reality there is a whole lot of control going on. In the case of impulsivity, the objective compulsivity is foregrounded: there is a strong sense of control but in reality there is no control.

The combination of obsessionality and compulsivity makes OCD a unique disorder. Obsessionality and compulsivity occur in obsessions and compulsions but manifest differently. In obsessions, obsessionality is passive and compulsivity direct, as for compulsions; obsessionality is active and compulsivity indirect. Yet one important aspect is missing: to describe the "nature" of OCD. OCD is not a static disorder but rather is a dynamic process in which the symptoms change after some time. In this process the attitude of the patient towards the obsessive-compulsive symptoms, the way they are reflected, plays a crucial role.

## Reflection

Contrary to many other psychiatric disorders, the OCD patient has an active and maintaining part in the development and the course of the disorder. Obsessions and compulsions always bring about something for patients as a result of which they are never without engagements. They arouse anxiety, fear, and loss of control, guilt, shame or aggression and they will be denied, resisted, avoided, doubted, smoothed over, compared, balanced. With OCD there is always a moment of subjective reflection as a result of which a viewpoint will be taken against the contents and the form of the symptoms. The patient with OCD is engaged in a dialogue with his or her disease and constantly reviews his or herself with respect to the contents and the form of the thoughts and acts. The development and evolution of OCD will be perpetuated and determined by this interaction. Subjective reflection is characteristic for OCD and expresses itself in crucial concepts such as egodystonia and insight, which often are used as decisive diagnostic criteria to distinguish OCD from other disorders.

Egodystonia means that a behavior, a desire, a dream, an impulse or a thought, is not recognized as your own or is in conflict with your self-image, in contrast to egosyntonia which expresses the harmony between an experience and our self-image. Since egodystonia refers to the self-image and the self-image is an imaginary creation, egodystonia does not necessarily touch on the reality or the truth. For example, the common obsession of being afraid of being homosexual can be egosyntonic for a true heterosexual who considers himself a homosexual and egodystonic for a true homosexual who considers himself a heterosexual. Egodystonia is a mismatch between an experience and a self-image. Because the self-image is an illusionary representation, rooted in a fictitious world and depending on various circumstances, egodystonia is in essence a variable condition. It should therefore be approached carefully as a conclusive differential diagnostic criterion for OCD.

Yet, egodystonia is a useful concept to differentiate OCD from obsessive-compulsive personality disorder (OCPD). Patients with OCPD do not have obsessions and compulsions, but rigidly force their will upon the world and the others. Patients with OCPD do not experience the rigid way they handle reality as disturbing, because it does not deviate from their self-image. The patient with OCPD refuses to take distance from his or her desire to manipulate the world to their own inflexible view. The demand for accuracy, precision and perfection synchronizes with their self-image and therefore is egosyntonic.

Equally, obsessions often are distinguished from delusions because delusions are egosyntonic. In delusions the patient thinks *with *the delusion, in obsessive-compulsive disorder, the patient thinks *against *the obsession. Actually, a delusion cannot be egosyntonic nor egodystonic because the patient does not have the ability to reflect upon the delusion. There is no match or mismatch between experience and self-image because the content of a delusion can never be taken as an object of reflection by a delusional patient. Delusions are prereflexive phenomena; they precede the moment of subjective reflection.

Another aspect related to subjective reflection is insight into the exaggerated and unreasonable nature of the obsessive-compulsive symptoms. Like egodystonia, insight is variable and difficult to assess unambiguously in OCD patients, making it less valuable as a strictly diagnostic criterion. Insight rarely occurs at the peak of the obsessional preoccupation. A patient suffering from contamination fear explains the following thought process: "When I visit the lavatory I imagine how filthy it would be to drink the toilet water. Then I think I could drink it, and then that perhaps I've already been drinking it, and then I'm certain I've been drinking it, then I doubt if I know for certain that I have been drinking it and finally I want someone to show me that I certainly have not been drinking it." During obsessionality and anxiety, OCD patients are no longer able to distinguish possibility and reality, between thought and act. But even in the presence of a professional health carer it can be hard to recognize the unreasonable character of a symptom through the strengthening impact of the ritual. A third grade student veterinary medicine explains that he is afraid that his parents might die. Every day he hangs out his shirt 15 times in a ritual order on a cloth hanger in the closet so that nothing will happen to his parents. When during consultation he is asked if he really thinks that there is a connection between his shirt and the death of parents, he answers the following. "My rational side says for fifty percent that there is not and my emotional side says for fifty percent that there is". "And besides", the student continues, "It works, because both my parents are still alive". Rituals are extremely powerful enforcers. The moment that we suspect that we can control uncontrollable life events like death, love, sexuality and health with a simple magical act, it will be difficult to take distance from the conviction that rituals are effective. Executing a simple magical act cannot be compared to taking the chance of letting people die, losing your loved ones, or getting sick. For security and control we easily can give up our rationality.

Apart from the insight into the obsessive-compulsive symptoms it is quite striking that the truth is not so important for an OCD patient, but how the truth is dealt with. A man explains that he thinks his father will get cancer if he often thinks about cancer himself. He is terrified to think of cancer unexpectedly or to be confronted with the word, convinced of causing cancer to his father. When the doctor confirms that scientific research really has proven that the chance of getting cancer increases when a family member thinks about it, the patient reacts full of disbelief. Truth and certainty are of another order.

## Certainty

Obsessions and compulsions can cover endless different themes. Some patients are afraid of numbers like 3, 5, 36, or 48, some are afraid of wishing their family members getting AIDS, afraid to become bold, or to realize that they can regulate their breathing. Others think that they gave their dog too hot a bath, are convinced of having once sold their child, or will get angry when somebody coughs. Others are obsessed by a strange taste of metal in their mouth, by the idea that their eye sight is not good enough, by the thought that the world looks different in reality, or that they could look like their mother. Others are obsessed with a large loose rock down a river in the south of France that could fall on someone's head, or with the precise functioning of language such as the ultimate meaning of the word "being". Others secretly organize the trash in the trashcans of their neighbourhoods, phone TV producers of children's shows to ask whether it was not a poisonous green frog that was on the show, refuse to enter villages where somebody ever committed suicide, or climb up a tree in autumn to cut off all the leaves to make it all perfect.

There is an endless variety of symptoms and in the past decade there have been many attempts to narrow down the heterogeneity of OCD to exclusive subtypes, each with a separate cause, demography, course and treatment. Without intending to question the value of this research strategy, it is important to realize that OCD symptoms also have a lot in common. Almost all OCD themes can be narrowed down to the problem of certainty and control. In the above-mentioned case the patient is admittedly anxious about the death of her daughter, but in particular about the *possibility *that she could kill her daughter, or - otherwise yet - for the inability to know with certainty that she will never kill her. Nobody, not even the patient herself, can offer the full guarantee that she could never kill her daughter.

In some cases the desire for absolute certainty is very explicit. A woman comes to our ward obsessed by the idea that her now adult son once at the hospital had been accidentally swapped with another child. To cure her from her obsession a genetic test had been performed three times. The test indicated every time that her son was indeed her own child. But the doctor was honest enough to report that there is an option of less than 0.00001% chance that the test result still might be wrong. The obsession continued unabatedly, because there was no absolute security. An elderly man doubted his entire life whether the driving examiner filled out the right score on his test form. He passed his test, but thought he scored too little points. After 27 years the man could not bear it any longer. He visited his driving examiner and asked him the score on test form to get certainty.

Though certainty is not always prominently present, it can be recognized in almost every obsession. Patients suffering from contamination fear are not so much afraid to become ill, but of the uncertainty that they could be contaminated. Patients who think they could push someone in front of the train at the station, or think that they could hit someone on the highway, are not so much afraid of the consequences but are afraid of the uncertainty of not knowing whether they will or will not. Patients with hoarding who find it hard to throw anything at all, want to make certain that they will not miss out on anything that they would ever be able to use. Obsessive-compulsive symptoms develop in a domain where there is no certainty what so ever and one is bound to lose control: sexuality, sickness, danger, death, and love.

Of all people, it is the patient with OCD that is continuously confronted with loss of control by the symptoms of his disorder, which create a need for absolute control and a desire for absolute security. The tragedy of certainty is that it is not based on objective knowledge or on external reality, but on a feeling. One can be totally wrong and still feel certain; one can be right and yet be in doubt. In the same individual a child can recognize with certainty Santa and his or her dad the schoolteacher with certainty the schoolteacher. Because knowledge is no guarantee for security, absolute certainty can never be gained. In the end, certainty always is the end of a process of acceptance; it is a feeling that is founded on the faith that it will be certain. The feeling of control and security comes at times when we are prepared to assume that there is control and certainty. Control and certainty are an illusion we have to believe in at times to safeguard us from being aware of the volatile nature of life and as well have to doubt it at times to live up with reality. This is not only a challenge for patients suffering from OCD, but an ongoing endeavour for all of us.

## Conclusion

This paper wanted to offer another perspective on obsessive-compulsive disorder through a phenomenological approach. In the proposed temporary and provisional analysis, the core phenomena of obsessive-compulsive disorder are characterized by obsessionality, compulsivity, and subjective reflection. In obsessionality the patient is preoccupied by a specific thought or act. Obsessionality is present in both obsessions and compulsions. One may speak of *"passive obsessionality" *during obsessions because the patient is submissively being obsessed and of *"active obsessionality" *during compulsions because the patient actively takes something into obsession. In compulsivity the patient feels compelled to think a specific thought or to perform a specific act. Compulsivity is present in both obsessions and compulsions. In an obsession, compulsivity is *direct*, because it is experienced from the beginning and it coincides with obsessionality. In a compulsion, compulsivity is *indirect *because it occurs only after a lap of time and is the result of a typical process. In the subjective reflection, the patient perpetuates and determines the course of the obsessive-compulsive symptoms. The theme of obsessive-compulsive disorder can be largely traced back to a desire for absolute certainty and control. The pathology of OCD delicately exposes the fragility of free will, and the desire for absolute certainty and control, which is embedded in all of us, either normal or abnormal.

All cases in this manuscript are hypothetical, and/or adapted from prototypical cases. All names and important identifying information have been removed and/or modified to insure patient anonymity.

## Competing interests

The authors declare that they have no competing interests.

## Authors' contributions

Damiaan Denys is responsible for the full content of the manuscript.

## References

[B1] Maurice Merleau-Ponty Phénoménologie de la perception 1945 Paris: Gallimard

[B2] Phenomenology online http://phenomenologyonline.com/

